# More than Mere Numbers: The Impact of Lethal Control on the Social Stability of a Top-Order Predator

**DOI:** 10.1371/journal.pone.0006861

**Published:** 2009-09-02

**Authors:** Arian D. Wallach, Euan G. Ritchie, John Read, Adam J. O'Neill

**Affiliations:** 1 School of Earth and Environmental Sciences, The University of Adelaide, Adelaide, South Australia, Australia; 2 School of Marine and Tropical Biology, James Cook University, Townsville, Queensland, Australia; 3 School of Earth and Environmental Sciences, The University of Adelaide, Adelaide, South Australia, Australia; 4 C&A Environmental Services, Rangeland Research and Restoration, West Burleigh, Queensland, Australia; Monash University, Australia

## Abstract

Population control of socially complex species may have profound ecological implications that remain largely invisible if only their abundance is considered. Here we discuss the effects of control on a socially complex top-order predator, the dingo (*Canis lupus dingo*). Since European occupation of Australia, dingoes have been controlled over much of the continent. Our aim was to investigate the effects of control on their abundance and social stability. We hypothesized that dingo abundance and social stability are not linearly related, and proposed a theoretical model in which dingo populations may fluctuate between three main states: (A) below carrying capacity and socially fractured, (B) above carrying capacity and socially fractured, or (C) at carrying capacity and socially stable. We predicted that lethal control would drive dingoes into the unstable states A or B, and that relaxation of control would allow recovery towards C. We tested our predictions by surveying relative abundance (track density) and indicators of social stability (scent-marking and howling) at seven sites in the arid zone subject to differing degrees of control. We also monitored changes in dingo abundance and social stability following relaxation and intensification of control. Sites where dingoes had been controlled within the previous two years were characterized by low scent-marking activity, but abundance was similar at sites with and without control. Signs of social stability steadily increased the longer an area was allowed to recover from control, but change in abundance did not follow a consistent path. Comparison of abundance and stability among all sites and years demonstrated that control severely fractures social groups, but that the effect of control on abundance was neither consistent nor predictable. Management decisions involving large social predators must therefore consider social stability to ensure their conservation and ecological functioning.

## Introduction

The long-term survival and ecological functioning of socially complex species such as wolves (*Canis lupus*) may depend on more than merely their numerical status [1]–[8]. The stability of their social units (packs) may be as important as their population size, but often only the latter is considered [1]. Wolves are eusocial [9], with breeding restricted to the dominant pair in the pack (alphas), while the other pack-members assist in rearing the young [7]. Young wolves have a long period of parental dependency, which provides the basis for the transfer of complex information between generations [1], [10]. Under natural conditions wolf-packs may show extraordinary stability. For example, Haber [1] reported on a wolf-pack that retained a distinct family lineage for over half a century, and a female that maintained alpha status for over 13 years until she died naturally at 18 years old. Few such examples are known however, due to the high level of human intervention in wolf populations. Many profound implications of wolf control remain largely invisible when only numbers are considered.

The control of wolves fractures their social structure, which may lead to changes in age composition, group size, survival rates, hunting abilities, territory size and stability, social behavior, genetic identity and diversity (reviewed in [1]). Controlled populations tend to have a higher proportion of young, breeding pairs and litters, due to the loss of pack structure which regulates breeding [2]. Brainerd et al. [7] assessed the impacts of breeder loss on wolf-pack dynamics and found that packs often disperse following the loss of the alpha pair. They also found that pups have a higher chance of survival in persisting larger packs. Following control, territory boundaries dissolve, and dispersing individuals (floaters) immigrate into vacant areas [7], [11], [12]. Complex behaviors that are learned and developed within stable packs, such as cooperative hunting techniques, may be lost, leading to simplification and aberration of social traditions [1].

The dingo (*C. l. dingo*) was introduced to Australia about 5,000 years ago possibly by Asian seafarers [13] and became established over the whole of the mainland. Replacing the thylacine (*Thylacinus cynocephalus*) and Tasmanian devil (*Sarcophilus harrisii*) [14], the dingo is now the largest terrestrial mammalian predator in Australia. Although dingoes differ from wolves in that they underwent a period of semi-domestication prior to their arrival in Australia [13], they are distinct from domestic dogs and display biological, behavioral and ecological traits characteristic of other wolf species [11], [15]–[18].

Since European occupation, dingoes have been targeted for lethal control over much of the continent, primarily because they prey on livestock [19]. Poison-baiting with sodium monofluoroacetate (1080) is the most common method of control [20]. The Dingo Barrier Fence (DBF), the world's longest man-made construction, spanning over 5,000 km, was built with the intention of eradicating dingoes from the southern parts of Australia where sheep-farming is common. Along the South Australian section of the DBF a 10–30 km buffer zone is intensively baited on the northern side to reduce the threat of reinvasion. Most pastoral stations north of the DBF also control dingoes because they are considered a threat to cattle [19].

Despite their keystone role as top-order predators [21], dingoes are also controlled in many conservation-designated areas. They are directly targeted because of a common belief that predator control will assist the recovery of threatened species [20], and to reduce their impact on neighboring pastoral stations. Dingoes are also indirectly affected because 1080 poison-baiting is extensively used to control red foxes (*Vulpes vulpes*), cats (*Felis catus*) and wild dogs (*C. familiaris*) [20]. Secondary poisoning may also occur when rabbits and other herbivorous animals are poison-baited [22], which is a common practice in Australia [20].

**Figure 1:**
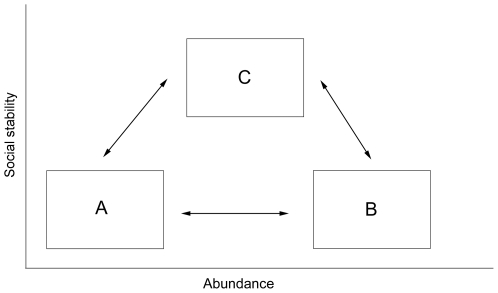
Theoretical model of the relationship between dingo abundance and social stability.

Nevertheless, dingoes remain abundant and they occur over much of mainland Australia, including areas inside the DBF [23]. Although they are remarkably resilient in the face of eradication efforts, very little is known about the effects of control on the integrity of their social structure. In this study we investigate the effects of dingo control on their abundance and social stability, and hypothesize that the two variables are not linearly related. We propose a theoretical model in which populations can fluctuate between three main states: (A) below carrying capacity and socially fractured, (B) above carrying capacity and socially fractured, or (C) at carrying capacity and socially stable (Fig. 1). We predict that control fractures pack structure and drives the population into unstable states (A or B) that are more likely to fluctuate with resource availability, and that relaxation of control allows the recovery of social stability and stabilization of population size (C).

## Methods

### Study sites

We assessed dingo abundance and territorial behavior at seven sites across the South Australian arid zone representing different management practices. At five sites dingoes have been controlled within two years of our field work, mostly with 1080 poison-baiting: Mungerannie (26°33'S, 139°42'E), Red Lake (southern area of Stuart Creek station; 30°11'S, 136°51'E), Andamooka (30°32'S, 137°05'E), Vulkathunha-Gammon Ranges National Park (GRNP; 30°29'S, 139°14'E) and Nantawarrinna (30°46'S, 139°02'E). There are very few places in the arid zone where permanent water is available and dingoes are not controlled [24], but after extensive surveys we located two sites: Pandie Pandie (26°33'S, 139°42'E) and Curdimurka (northern section of Stuart Creek station; 29°28'S, 137°03'E), where dingo control has been minimal for at least five years. Pandie Pandie, Mungerannie, Curdimurka and Red Lake are outside the DBF, while Andamooka, GRNP and Nantawarrinna are inside the DBF. Study sites were 200–500 km^2^, and each included at least three permanent water sources.

Red Lake, Andamooka, Nantawarrinna and Curdimurka were surveyed more than once to study the effects of management changes on dingo abundance and social stability. At Red Lake dingo control was relaxed, in Andamooka and Nantawarrinna dingo control intensified, and at Curdimurka there were no management changes and dingoes were generally not disturbed. Red Lake is inside the buffer zone and was baited annually until 2006. We surveyed this site in 2006 and for two years (2007, 2008) following relaxation of control. Rainfall was below average during the study period (www.bom.gov.au). Red Lake borders Andamooka station and the two sites are separated by the DBF. At Andamooka dingoes have been controlled with shooting and sporadic low-intensity baiting between 2001 and 2007. In May 2008 Andamooka was subjected to an intensive poison-baiting treatment and we monitored this site several months before (October 2007) and after (October 2008) this event. Conditions were favorable in both years following an above-average rainfall event in early 2007 (www.bom.gov.au), which increased prey availability (Wallach unpublished data). Nantawarrinna has a long history of dingo eradication efforts, but since the station was de-stocked in the 1990's, dingo control has been conducted irregularly. In mid 2008, Nantawarrinna was subjected to an intensive poison-baiting treatment and we monitored this site several months before (December 2007) and after (November 2008) this event. Rainfall was below average during the study period (www.bom.gov.au). Curdimurka was surveyed in November 2007 and September 2008, and rainfall was below average (www.bom.gov.au).

### Relative abundance

Relative abundance of dingoes was assessed by the passive track survey method described previously in Wallach et al. [23]. In short, relative density (R_de_) was determined by dusting randomly located 500-m transects (at least 1 and 2 kms apart for off and on road transects, respectively) and counting the number of dingo crossings over three days, giving an average value of tracks/500 m/day (9–25 transects/site). Transects were located both on unformed dirt roads and off roads, where possible. We also estimated the relative distribution (R_di_) of dingoes (proportion of the study site occupied) by recording the presence or absence of fresh dingo tracks in random 2-ha plots scanned for 30 min (21–39 plots/site). An Index of Abundance (IA) was calculated as follows: 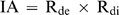



### Social stability

Intensive persecution has taught many dingoes to avoid human contact [23] hence opportunities for direct observations of wild dingoes are limited. Furthermore, any attempt to capture animals for radio collaring may bias the sample toward naïve floaters. Our first aim therefore was to develop an indirect method of assessing social stability. We surveyed the frequency of social stability indicators, scent-marking and howling, under differing degrees of control.

Scent-marking with scats, urine and ground-rakings are well known forms of social communication in canids [e.g. dingo: 16], [17; other wolves: 12], [Bibr pone.0006861-SilleroZubiri1] [25]. Scent-marking communicates pack size and composition, individual social and breeding status, and is used to advertise ownership and territory boundaries [12]. A reduction in scent-marking following the breakdown of a wolf pack (due to control or disease) may be followed by a rapid shift in territory boundaries and infiltration of floaters [12]. Like scent-marking, howling communicates a diversity of messages such as identity, location, age, size, aggressiveness, social and breeding status, and pack size and composition [dingo: 16], [17; other wolves: 12], [26], [Bibr pone.0006861-Harrington1] [27]. Howling and scent-marking are both more common among pack members than among floaters [dingo: 17; other wolves: 28], [Bibr pone.0006861-Rothman1] [Bibr pone.0006861-Nowak1] [29; coyote *C. latrans*: 30].

We surveyed dingo scat abundance and location to assess scent-marking intensity, because annual variation in deposition rates is small [12], [17], [25], detectability is high, and in our study sites scats normally endure for roughly 3–6 months, although some persist much longer (Wallach unpublished data). Scats are long-term visual cues, especially in low rainfall regions, and are placed to maximize their visual effect [31]. They are often found concentrated at distinct focal points such as road junctions, elevated objects and carcasses [12], [17], [32]. The positioning of scats on conspicuous objects can assist in the identification of canid territorial marking [33]. Hence we consider the strategic deposition of dingo scats in prominent locations, relative to randomly located scats, to be an indicator of territoriality and pack stability.

The locations of 890 dingo scats (122 [SE 48] scats/site), mostly from outside the DBF, were recorded while surveying the 2-ha plots (described above), to determine the degree to which scats were placed randomly or at focal points. Focal points that could potentially provide a resource are referred to as resource points. A scat was considered to be a scent-mark if it was deposited on a distinct focal point, was part of a latrine, or if it was found on a conspicuous object. We then conducted a scat survey of the most prominent focal points at each site (average of 80 focal points/site) to determine the level of scent-marking activity. We recorded all dingo howling events at the study sites that we camped in for a minimum of two weeks (all sites apart from the GRNP). We also estimated the age of dingoes (whether young: <1 year, or mature: >1 year) based on their size and appearance, during occasional clear, direct observations.

### Statistical analyses

We compared scent-marking at different resource points with a Kruskal-Wallis test, and we tested the relationship between scent-marking and howling activity with a Spearman's rank correlation test. A site was considered ‘controlled’ if baiting or shooting had been conducted within the past 2 years. We compared abundance and scent-marking between sites with (N = 5) and without (N = 2) control using a Mann-Whitney U-test, and we used the average of multiple years for sites that were surveyed more than once. A Chi-square test was used to compare the number of young and mature dingoes in controlled and non-controlled sites. A best-fit regression analysis was employed to assess the relationship between scent-marking, howling and abundance with the time elapsed since control, between scent-marking and abundance, and between howling and abundance. We also compared dingo control intensity, scent-marking, howling and abundance with both average annual rainfall and recent rainfall (accumulation of one year prior to the study). Control intensity was quantified as the maximum time any of our study site was not controlled (standardized at 6 years), minus the time elapsed since control was applied at each site. When comparing the change in abundance and scent-marking following management changes we used a Mann-Whitney U test because some of the samples (e.g. resource points and transect location) were not tied even though the sites were.

We constructed and compared generalized linear models (using a Poisson distribution and log link function) of dingo scent-marking and howling with the Akaike's Information Criterion (AIC). We used an information-theoretic approach (ITA) and constructed all possible configurations (best subsets) of independent variables that may predict each response variable. We compared the support for models according to differences in their AIC scores [34], as well as calculating Akaike model weights (*wi*) [35]. We retained all models which were within a 95% confidence set [33]. The relative importance of predictor variables was calculated by summing *w_i_* across all models in which the variables occurred. The candidate models included: (i) time elapsed since control, (ii) poison-baiting frequency (baiting/year), (iii) distance from human activity centers (e.g. towns, camping grounds), (iv) dingo abundance, and for howling only: (v) breeding season (between April-August, following [16]). Howling frequency was arcsine-transformed prior to analyses in accordance with recommendations in Quinn and Keough [36].

## Results

The location of dingo scats found on the 2-ha plots was not random, and 97% (N = 890) were found on clearly defined focal points; including water points, rabbit warrens, carcasses, trees where eagles nested and fed, roads, gates and isolated trees. Scats were often deposited on conspicuous objects and were part of latrines. Water points, rabbit warrens, carcasses and eagle nests were the most common and intensively scent-marked resource points, and up to 80% were scent-marked in a given area. Dingoes scent-marked water points (12.84 [SE 3.22] scats/water point; N = 69) more intensively than rabbit warrens (1.32 [SE 0.23] scats/warren; N = 555), carcasses (0.89 [SE 0.27] scats/carcass; N = 204) or eagle nests (3.64 [SE 1.25] scats/nest; N = 32) (Kruskal-Wallis: H = 39.76, d.f. = 3, p<0.001).

Scent-marking and howling frequency were not significantly correlated (Spearman (*r*_s_) _0.05 (2), 10_  = 0.23, p = 0.52), and were predicted by a different set of variables (Table 1). Scent-marking activity was explained by four models that included four variables. Time since control was by far the most important and positive predictor of scent-marking. Relatively weaker predictors were dingo abundance, poison-baiting frequency and distance from human activity (Table 1). Conversely, howling frequency was explained by seven models that included four variables. Distance to human activity was the strongest predictor of howling (howling frequency increased as distance from human centers increased); followed by time since control, poison-baiting frequency and timing of the breeding season. Dingo abundance did not feature in any of the top-models predicting howling (Table 1).

**Table 1:** Generalized linear models of dingo scent-marking and howling, using best subsets (AIC).

Model			AIC	Δ*_i_*	*w_i_*		Variables	Importance *w_i_* (direction of effect)
Scent-marking	Time since control		22.18	0.00	0.44		**Time since control**	**1.00 (+)**
	Time since control	Dingo Abundance	23.80	1.62	0.20		Dingo Abundance	0.20 (+)
	Time since control	PBF	23.83	1.64	0.19		PBF	0.19 (−)
	Time since control	Human distance	24.07	1.89	0.17		Human distance	0.17 (−)
Howling	Human distance		11.02	0.00	0.21		**Human distance**	**0.51 (+)**
	Time since control		11.22	0.20	0.19		Time since control	0.30 (+)
	PBF		11.56	0.54	0.16		PBF	0.27 (−)
	Breeding season		11.92	0.90	0.14		Breeding season	0.22 (+)
	Time since control	Human distance	12.37	1.36	0.11			
	PBF	Human distance	12.46	1.44	0.10			
	Human distance	Breeding season	12.98	1.97	0.08			

Only models which are within the 95% confidence set for each model set are shown.

Δ*_i_* = model score differences, *w_i_* = Akaike model weights. Variables with importance (*w_i_*) greater than 0.5 are shown in bold, and the direction of effect are indicated in brackets. PBF = Poison-baiting frequency, Human distance = distance from centers of human activity.

**Figure 2:**
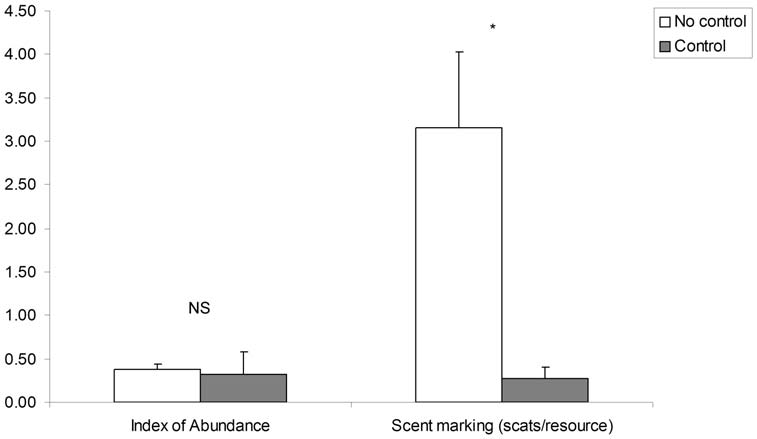
Comparison of abundance and scent-marking between sites with and without dingo control (average + se).

Sites where dingoes had been controlled within the previous 2 years were characterized by low scent-marking activity (Mann-Whitney Z = 1.97, p<0.05; Fig. 2), but abundance was similar between sites with and without control (Mann-Whitney Z = 1.16, p = 0.25; Fig. 2). Dingo abundance was however reduced at sites that had been poison-baited within the past 3 months (Mann-Whitney Z = 2.32, p<0.05). Of 15 dingoes observed in the controlled sites, 93% were young (N = 15), while 75% of dingoes observed in the non-controlled sites (N = 20) were mature (χ^2^ = 16.13, d.f. = 1, p<0.0001). Scent-marking and howling increased linearly the longer an area was allowed to recover from control (scent-marking: R^2^ = 0.94, d.f. = 10, p<0.001, howling: R^2^ = 0.46, d.f. = 9, p<0.05), but we found no significant relationship with dingo abundance (p>0.05). Dingo control intensified and scent-marking decreased as average annual rainfall increased (control intensity: R^2^ = 0.38, d.f. = 9, p<0.05, scent-marking: R^2^ = 0.39, d.f. = 9, p<0.05), while no significant effect was found for howling, abundance or recent rainfall.

**Figure 3:**
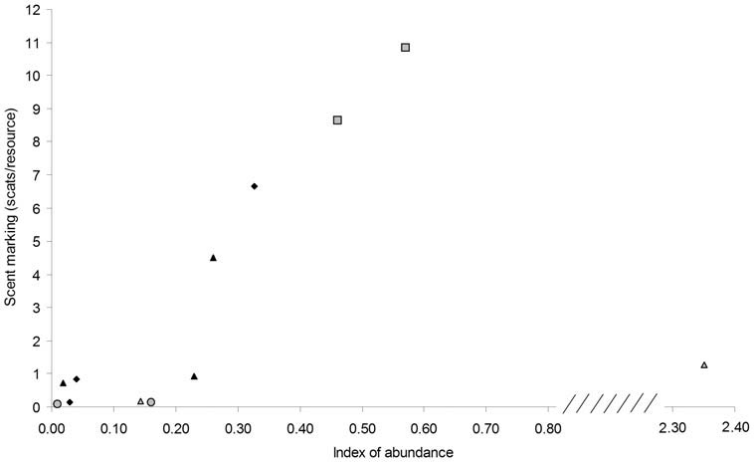
Relation between dingo abundance and scent-marking activity. Black triangles: Red Lake (2006, 2007, 2008), grey triangles: Andamooka (2007, 2008), grey circles: Nantawarrinna (2007, 2008), grey squares: Curdimurka (2007, 2008), and black diamonds represent Pandie Pandie, Mungerannie and the GRNP.

The relationship between dingo abundance and scent-marking followed a bell-shaped curve (quadratic best fit: R^2^ = 0.84, d.f. = 9, p<0.001; Fig. 3). Scent-marking activity was reduced at low densities and began to increase exponentially at IA = 0.2. One site (Andamooka in 2008) had a particularly high dingo abundance index but scent-marking was low, corresponding with the prediction of a state B scenario illustrated in Fig. 1. Although our data is mostly restricted to the left side of the curve (Fig. 3), we found that the quadratic best-fit line was also supported over a linear line after removing Andamooka 2008. Using AIC, we found that a model with the variable ‘abundance’ (linear) + ‘abundance squared’ (quadratic) was substantially better supported (>1000 times) than a model with just ‘abundance’ (linear). Adding this quadratic variable did not change the relative importance of abundance on the scent-marking model in Table 1. Howling frequency followed a similar pattern but was considerably more variable (p>0.05; data not shown).

**Figure 4:**
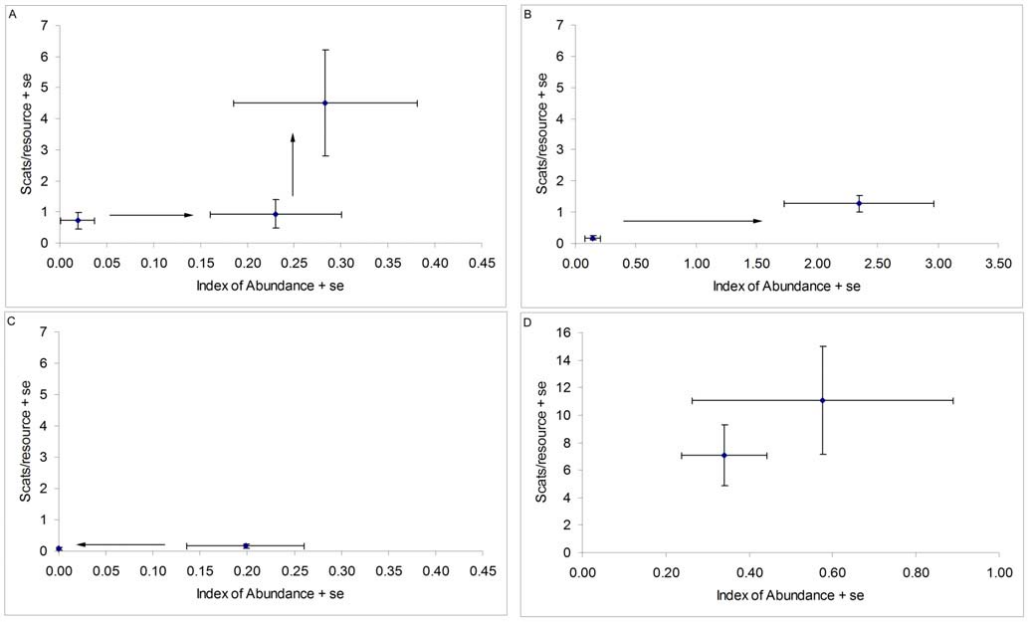
Effect of relaxation or intensification of control on dingo abundance and scent-marking behavior. (a) Relaxation of control (Red Lake 2006–2008); (b) intensification of control during a productive period (Andamooka 2007–2008); (c) intensification of control during a dry period (Nantawarrinna 2007–2008); and (d) no control in both years (Curdimurka 2007–2008). Arrows denote the direction of meaningful significant changes between the years.

One year after the cessation of poison-baiting in Red Lake, dingoes increased in abundance (Mann-Whitney Z = 2.37, p<0.05) but scent-marking remained low (Mann-Whitney Z = 0.49, p = 0.62; Fig. 4a). After two years abundance stabilized (Mann-Whitney Z = 0.38, p = 0.71) and scent-marking increased significantly (Mann-Whitney Z = 6.35, p<0.001; Fig. 4a). Also after two years the first howl was heard (N = 14).

Poison-baiting at Andamooka, coupled with high resource availability, resulted in a 16-fold increase in abundance (Mann-Whitney IA: Z = 2.8, p<0.01; Fig. 4b), bringing the index of abundance to the highest level detected in this study (see also Fig. 3). The size of the tracks and the location of an active den indicated that many of these dingoes were young. This change in abundance was also followed by an increase in scent-marking (Mann-Whitney: Z = 5.22, p<0.001; Fig. 4b), but after correcting for relative abundance, scent-marking was found to have decreased (Mann-Whitney scent-marking/IA: Z = 4.33, p<0.001). No howling events were recorded in either year.

In 2007 dingoes were relatively abundant at Nantawarrinna but incidence of scent-marking was low (N = 18). After Nantawarrinna was poison-baited, dingo abundance dropped to almost zero (Mann-Whitney IA: Z = 2.86, p<0.01; Fig. 4c) and scent-marking remained low (N = 4). Howling was relatively common at Nantawarrinna in 2007 (18% of nights (N = 28), with up to four dingoes howling together). A single dingo howled in 2008 (N = 14).

Curdimurka was not subjected to dingo control, and abundance and scent-marking activity remained high and relatively stable, although there was a trend of increase for scent-marking (Mann-Whitney IA: Z = 0.41, p = 0.68, scent-marking: Z = 1.72, p = 0.09; Fig. 4d). Howling frequency was also similar between years (14.29% (N = 14) and 21.43% (N = 14) of nights in 2007 and 2008, respectively).

## Discussion

**Figure 5:**
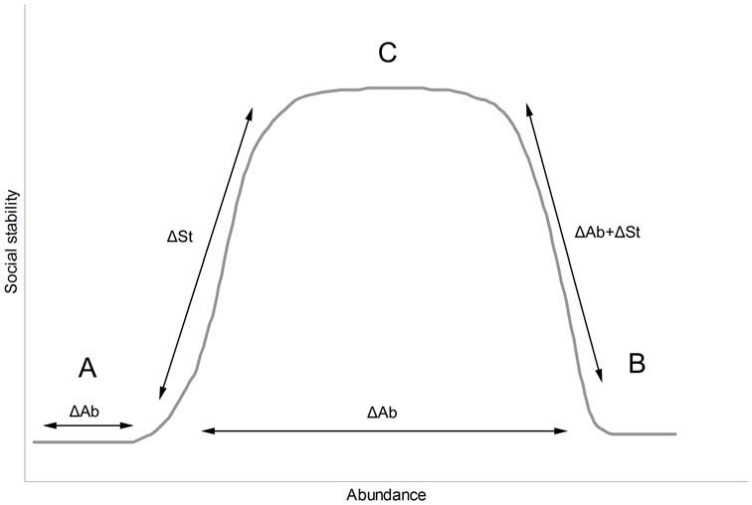
Influence of management on the abundance (Ab) and social stability (St) of dingoes.

Our study provides evidence that the relationship between dingo abundance and social stability is not linear, but may rather follow a bell-shaped curve. Lethal control systematically fractures social units and releases population abundance to bottom-up processes, which drives population size in either direction. Fig. 5 illustrates the effect of management on the dynamic relation between dingo abundance and social stability as indicated in this study. Under the influence of control, dingo populations may be driven towards the unstable states of A or B. The changes following relaxation of control indicate that recovery may follow two main phases. Populations recovering from state A will first increase in abundance, potentially driving the population into state B. The next phase is an increase in stability followed by a reduction in population growth rate (A to C) or size (B to C).

Researchers usually focus solely on abundance, probably because assessing social stability may not always be feasible, due to constraints on research time and resources. This study used a rapid and non-invasive method of assessing social stability that requires efforts similar to those used in estimating abundance. Sites where dingoes have been undisturbed for several years are characterized by consistent scent-marking of available resource points. In the arid zone, water points, carcasses, active warrens or dens, nests and roads are the most common focal points of scent-marking activity. Our stable study sites had an average of 6–10 scats per resource point (Fig. 3), but some scent-posts had over 100 scats. Where dingoes are stable and fear of humans is minimal, howling is heard on most nights and most frequently during the breeding season [16]. Sites that are subjected to dingo control are easily recognized by the paucity of scent-marking, whether population abundance is low or high. Scent-marking appears to be the most consistent indicator of social stability because it can be reliably predicted by the occurrence of control. While control also predicts howling frequency, proximity to centres of human activity is the strongest predictor (Table 1). These differences explain why howling and scent-marking are not always correlated. The importance of scats in canid scent-marking, and their utility as indicators of stability, is probably more applicable in low rainfall regions where scats can endure as visual cues for extended periods of time.

Another observable symptom of pack disintegration appears to be an increase in attack rates on livestock. Allen and Gonzales [37] provided experimental evidence suggesting that calf losses are higher where dingoes are baited than where they are left undisturbed. They found, as we did, that baiting does not always reduce dingo numbers. A similar pattern was found at Pandie Pandie (no control) and Mungerannie (annual poison-baiting). We found no signs of dingo predation on cattle at Pandie Pandie (N = 56), while at Mungerannie 14% (N = 44) of carcasses were calves, and all appeared to have been killed by dingoes (Wallach & O'Neill unpublished). Similarly, several weeks after Nantawarrinna was poison-baited in 2008, a neighbouring property lost 24 of 30 sheep to dingoes in one day. The sheep were killed but not eaten (T. Coulthard, personal communication). Long-term data on coyote control also indicate that control does not significantly reduce livestock predation [38] nor does it improve production [39].

In the course of this study, we did not find a single place where dingoes had clearly reached state C. Even at Pandie Pandie and Curdimurka, dingoes have occasionally been shot or poisoned. Although abundance, scent-marking and howling did not change significantly in Curdimurka, there was a trend of increase (Fig. 4d), indicating that dingoes may still be recovering from past control. Similarly, the regression analysis for scent-marking and howling as functions of time since control were linear and did not plateau, although this may be a result of the small sample size. The lack of social cohesiveness in dingo populations appears to characterize the vast majority of Australia. In the arid zone there is a trend of dingo control intensification, and social destabilization, as average annual rainfall increases. Thus, the more potentially productive areas are the most highly controlled against dingoes. The implications of such widespread control are largely unknown, but probably result in reduced fitness and impaired ecological functioning at a continental scale.

Eusocial systems have developed to increase fitness for pairs that are part of multi-generational groups comprised mainly of non-breeding helpers [9], [10]. Following control, the remaining individuals may be subjected to reduced survival rates by creating populations with many lone breeding pairs [7]. Dingoes, like other wolves, are cooperative hunters, and their hunting abilities are directly related to pack size, age and experience [40], [41], [42]. Control-related fitness costs may also be indirect. For example, reduced group size may increase the loss of kills to scavengers [6], and social fracturing may induce chronic stress levels in a population [43].

The long dependency period of many young social carnivores (e.g. wolves) attests to the vital role of learning within these species [1]. At Curdimurka we observed a dingo pup (approximately 4 weeks old) actively searching out rabbit warrens and buck heaps and dingo scent-posts for scent-marking. At Pandie Pandie a two-month-old dingo was heard howling daily with the same adult, presumably its mother, and often in chorus with three additional adults that howled regularly together. At Curdimurka we observed a dingo that washed his food. Two pieces of kangaroo meat (from an ant-covered carcass) were washed in a spring approximately 30 m from the carcass. Food-washing is often cited as an example of culture in primates [44]. Although we do not know how this behaviour developed, it is interesting to note that it occurred in one of the stable sites.

The role of learning is particularly evident in the case of dingoes surviving in the face of eradication efforts. For instance, inside the DBF in South Australia some national parks poison-bait fortnightly (S. Gillam unpublished data), with poison-baits often distributed by aircraft, achieving extensive coverage of large areas that are otherwise inaccessible (Bounceback unpublished report). Despite this, Wallach et al. [23] located dingoes surviving in areas deep inside the DBF, near towns and sheep farms, that have avoided detection for several decades, and found that scent-marking rates were relatively high. Although pack stability is usually disrupted under control, dingoes surviving under conditions of intensive persecution must have retained stability, because survival depends on specialized skills (avoiding contact with humans, livestock predation, and baits) that must be passed on to their offspring.

Hybridization with dogs is considered one of the main threats to dingo survival in Australia, spurring the Victorian State Government to shift the dingo from the vermin list to the endangered species list in 2008. We believe that the rate of hybridization is a direct consequence of dingo control. Like all wolf species, dingoes are highly territorial and aggressive, and it is with great difficulty that outsiders join stable packs [dingoes: 17; other wolves: 12], [Bibr pone.0006861-SilleroZubiri1] [45]. Under natural conditions genetic lines are protected through kin selection, and genetic variation within packs may be small due to inbreeding and aggressive behavior towards outsiders [1], [46]. This can give rise to the development of genetic traits unique to each pack, such as coat color [dingo: 11; wolf: 1). Dingo control may increase the number of floaters which are more likely to breed with dogs [47]. Similarly, hybridization between wolves and dogs has been reported from Latvia where wolf-hunting was common [48]. We propose that the most efficient way to conserve the genetic identity of dingoes and other wolves is to cease control.

The dingo is the only large terrestrial mammalian predator in Australia, the next largest being the invasive red fox. Australia is now home to a diversity of large prey species, mostly invasive, that have successfully eluded eradication efforts. These include goats (*Capra hircus* 15–80 kg), feral pigs (*Sus scrofa* 25–175 kg), six species of deer (*Dama dama, Cervus* spp., and *Axis* spp. up to 300 kg), feral donkeys (*Equus asinus* 300–350 kg), feral horses (*E. caballus* ca. 500 kg), feral cattle (*Bos taurus* 500–900 kg) and camels (*Camelus dromedaries* 600–1000 kg) [49]. Dingoes may have the potential to regulate even the largest of prey (but see [50]), most likely mainly through risk effects [51], [52], but only under conditions of long-term pack stability can this be reliably tested.

As long as only numbers are considered, the full ecological benefits of dingoes will remain unknown. It is the *pack* that is the top predator, not the individual dingo. Without the pack, a dingo is functionally equivalent to a large fox. Australia has suffered the worst rate of mammalian extinctions worldwide [14] and this crisis is directly linked with dingo control [21]. The ecological role of the dingo as Australia's top predator has recently moved into the spotlight of research attention [18]. It is vitally important that future research considers the role of social stability, to ensure the conservation and ecological functioning of socially complex top-order predators.
